# Development of Delirium in the Intensive Care Unit in Patients after Endovascular Aortic Repair: A Retrospective Evaluation of the Prevalence and Risk Factors

**DOI:** 10.1155/2015/405817

**Published:** 2015-09-02

**Authors:** Yohei Kawatani, Yoshitsugu Nakamura, Yujiro Hayashi, Tetsuyoshi Taneichi, Yujiro Ito, Hirotsugu Kurobe, Yuji Suda, Takaki Hori

**Affiliations:** Department of Cardiovascular Surgery, Chiba-Nishi General Hospital, 107-1 Kanegasaku Matsudo-Shi, Chiba Prefecture 2702251, Japan

## Abstract

Delirium is an acute form of nervous system dysfunction often observed in patients in the intensive care unit. Endovascular aortic repair (EVAR) is considered a minimally invasive surgical treatment for abdominal aortic aneurysm. Although the operation method is widely used, there are few investigations of the rate and risk factors of delirium development after the operation. In this study, we retrospectively examined the rate of delirium development in the intensive care unit (ICU) after EVAR, as well as the associated preoperative risk factors and effects on the lengths of ICU and hospital stays. We examined the 81 consecutive patients who underwent elective EVAR between November 2013 and August 2014. The Intensive Care Delirium Screening Checklist was used to diagnose delirium. Twenty patients (24.7%) were diagnosed with delirium in this study. The ICU and hospital length of stays of patients with delirium were 3.3 ± 2.4 days and 14.5 ± 11.9 days, respectively, the latter of which was significantly longer than that of patients without delirium (*p* = 0.019). Additionally, renal dysfunction, preoperative benzodiazepine use, and intraoperative transfusion were found to be risk factors for the development of delirium after elective EVAR.

## 1. Introduction

Delirium is an acute form of nervous system dysfunction that is characterized by disturbed consciousness, inattention, and disorientation and is often observed in patients in the intensive care unit (ICU) [1]. The development of delirium after surgery can adversely affect a patient's outcome through prolonged hospital stays and increased complications [2]. Patients who develop delirium are at risk of self-removal of catheters or other tubes or of falling out of bed. Delirium prolongs stays in the ICU, increases the burdens on ICU staff members, and increases the incidence of complications during hospital stays [3–5]. Delirium can also affect a patient's relatives, as 70% of families of affected patients have reported feelings of distress [6].

The prevalence of delirium in the ICU varies greatly, with reports ranging from 16% to 80% [7–11]. Patient characteristics or comorbidities might affect this prevalence. For example, delirium is commonly reported in cardiovascular surgery or cardiovascular ICUs. In a study of delirium in the cardiovascular ICU, McPherson et al. reported a prevalence rate of 26% for both cardiac surgical patients in the cardiovascular surgery ICU and cardiology patients in the cardiovascular ICU [12]. Surgical procedures may also affect the development of delirium. Bucerius et al. examined the rate of delirium development after coronary artery bypass surgery and reported a lower rate after off-pump coronary artery bypass grafting (CABG), which is considered minimally invasive, compared to after on-pump CABG [13].

Endovascular aortic repair is considered a minimally invasive surgical treatment for abdominal aortic aneurysm, for which a cardiopulmonary bypass or major aortic vessel clamping is not required. Although the treatment method is widely used, no investigations of the rate and risk factors of delirium development after endovascular aortic repair (EVAR) have been conducted. Accordingly, in this study, we retrospectively examined the rate of delirium development in the ICU after EVAR, as well as the associated preoperative risk factors and effects on the lengths of ICU and hospital stays.

## 2. Methods

### 2.1. Patients

From among 171 consecutive cases involving endovascular aortic repair at a single institute (Chiba-Nishi General Hospital) between November 2013 and August 2014, 81 cases subjected to planned EVAR for abdominal aortic aneurysm were retrospectively examined. At our facility, all patients who undergo endovascular aortic repair are admitted to the ICU after surgery, upon recovery from anesthesia and complete mechanical ventilation. All patients included in this study were admitted to the ICU following elective EVAR and after extubation in the operation room. All patients undergoing surgery at our facility were given an explanation of the significance of publishing their clinical data and figures at academic meetings or in the scientific literature, and all patients who provided signed consent to participate in the studies in our facility are included.

### 2.2. Clinical Data

We retrospectively reviewed recorded patient characteristics such as age, gender, and medical history (e.g., hypertension, insulin-dependent diabetes, and cerebral infarction with residual paralysis) in the medical records. Preoperative factors such as left ventricular dysfunction, renal dysfunction, and preoperative benzodiazepine use were also investigated. Left ventricular dysfunction was defined as a left ventricular ejection fraction of <30% as detected by preoperative cardiac ultrasonography; renal dysfunction was defined as a serum creatinine level of >1.10 mg/dL for men or >0.80 mg/dL for women. All benzodiazepine use within 48 hours prior to the surgery was included in the analysis. Preanesthetic medication is not administered at our hospital; therefore, in this study benzodiazepine was used as an oral sleeping agent.

Intraoperative factors such as the surgical duration, amount of contrast media used, irradiation time, presence or absence of intraoperative packed red blood cell transfusion, and presence or absence of transcatheter arterial embolization were investigated. The investigated postoperative factors included the Acute Physiology and Chronic Health Evaluation II (APACHE II) score and the lengths of ICU and hospital stays.

### 2.3. Assessment

Each patient received care from 2 attending nurses. Each attending nurse regularly filled out the nursing record every 2 hours and logged any other findings. Additionally, the Intensive Care Delirium Screening Checklist (ICDSC) was used to diagnose delirium. In a previous prospective study, the pooled sensitivity and specificity of the ICDSC as a diagnostic tool were 74% and 81.9%, respectively [14]. Findings associated with the ICDSC checklist items, which include an altered level of consciousness; inattention; disorientation; hallucination, delusion, or psychosis; psychomotor agitation or retardation; inappropriate speech or mood; sleep-wake cycle disturbances; and symptom fluctuations, were recorded to the extent possible in order to generate a total score with which to evaluate the presence of delirium. ICDSC checklist items were reviewed in the ICU diaries of all included patients, and a score of ≥4 was considered to indicate the presence of delirium.

### 2.4. Statistical Analysis

Continuous variables are presented as means ± standard deviations, and categorical variables are presented as totals (percentages [%]). Continuous variables such as age, surgical duration, amount of contrast media used, APACHE II score, and lengths of ICU and hospital stays were analyzed using the Mann-Whitney *U* test. The chi-square test was used to compare categorical variables such as gender, medical history, presence or absence of intraoperative packed red blood cell transfusion, presence or absence of transcatheter arterial embolization of the internal iliac artery, and an APACHE II score of ≥16 points. Differences were considered statistically significant at a *p* value of <0.05. Risk factors with significant differences (*p* < 0.05) or those that tended toward a difference despite a lack of significance (*p* < 0.10) in a univariate analysis were subjected to a multivariate analysis using a stepwise multiple logistic regression model to determine the factor with the greatest impact. All statistical analyses were performed on a personal computer using the statistical software package SPSS for Mac (Version 22; SPSS Inc., Chicago, IL, USA).

## 3. Results

Eighty-one patients (mean age, 74.4 ± 7.9 years) met the inclusion criteria and were included in this study; of these, 83% were men. Twenty patients (24.7%) were diagnosed with delirium in the ICU after elective EVAR. Eight patients presented hyperactive type; 12 patients presented hypoactive type. The duration of delirium was 1.5 ± 0.83 days. Haloperidol was administered in 6 patients, risperidone was administered in 2 patients, dexmedetomidine was administered in 2 patients, and midazolam was administered in one patient.

Renal dysfunction (35% versus 11%, *p* = 0.016) and preoperative benzodiazepine use (30% versus 6.6%, *p* = 0.018) were more often observed in patients with delirium than in patients without delirium (Table 1). Regarding intraoperative/postoperative factors, a longer surgical duration (122 ± 41.7 min versus 102 ± 37.3 min, *p* = 0.047), more frequent intraoperative red blood cell transfusion (20% versus 1.6%, *p* = 0.003), and greater incidence of APACHE II scores ≥ 16 were observed among patients with delirium (35% versus 21%, *p* = 0.016) (Table 2). Whereas no significant difference was observed in the ICU length of stay (3.3 versus 2.5 days, *p* = 0.17), the hospital length of stay was longer in patients with delirium than in those without delirium (14.6 versus 9.9 days, *p* = 0.019). We did not observe in-hospital death in this study.

The results of a multiple logistic regression analysis revealed that comorbid renal dysfunction (odds ratio [OR] = 4.499, 95% confidence interval [CI]: 1.152–17.572) and preoperative benzodiazepine use (OR = 7.628, 95% CI: 1.670–34.843) were significant preoperative risk factors for developing delirium. Furthermore, intraoperative red blood cell transfusion (OR = 12.254, 95% CI: 1.113–134.881) was found to be a significant intraoperative/postoperative risk factor for delirium in the ICU (Table 3).

APACHE II is Acute Physiology and Chronic Health Evaluation II; CI is confidence interval; ICU is intensive care unit; and OR is odds ratio.

## 4. Discussion

The cardiovascular surgical procedure of endovascular aortic repair does not require clamping of the major aortic vessels or extracorporeal circulation and is therefore considered minimally invasive. Accordingly, in this study we examined the development of delirium in the ICU after elective EVAR among patients who underwent endovascular aortic repair. We found that 24.7% of these patients developed delirium in the ICU after elective EVAR. Interestingly, a similar previous study reported a delirium prevalence of 26% in both the cardiac surgery ICU and cardiovascular ICU [12].

Delirium has been reported to increase postsurgical mortality and prolong the lengths of ICU and hospital stays [5, 15, 16]. Similarly, in this study, the hospital stay was significantly longer for patients with delirium by a mean difference of 5 days. However, no significant difference in the ICU length of stay was observed between patients with and without delirium.

Some previous studies have reported renal dysfunction as a risk factor for postsurgical delirium development; for example, Martin et al. reported that renal dysfunction was a risk factor for delirium development after CABG [17]. Renal dysfunction is among the most important patient characteristics to investigate when performing EVAR. Similarly, the present study revealed that patients with renal dysfunction were 4.5-fold more likely to develop delirium and that renal dysfunction was therefore an important postoperative management factor. Contrast media, which is used multiple times throughout the treatment process, from the determination of an indication for EVAR to intraoperative angiography via graft sizing, is known to cause or exacerbate renal function, especially in patients with renal dysfunction. However, no association was observed between the amount of contrast media used and the development of delirium in this study.

In the present study, patients who received transfusions were 12.25-fold more likely to develop delirium, and therefore intraoperative transfusion had the greatest impact among the investigated risk factors. Lin et al. conducted a meta-analysis and reported that intraoperative transfusion was a risk factor for delirium development after cardiac surgery; the authors further stated that this risk factor might reflect the surgical duration or procedural complications [18]. However, elective EVAR, which was investigated in this study, is a standard surgical procedure with little intersurgical variation. Moreover, no association was observed between the surgical duration and delirium in a multivariate analysis. Nonetheless, transfusion was a strong risk factor for the development of delirium in this study, despite the lack of aortic perforation or changes from the planned surgical procedure to open surgery. In other words, transfusion was a strong risk factor, even if effects related to the operative procedure or other factors were minimal.

Although the mechanism of delirium development has not been fully elucidated, the causative factors can be divided into drug-related factors, patient-related characteristics, acute pathological conditions, and chronic pathological conditions [19]. For example, benzodiazepine use has been reported as a risk factor for delirium in some studies [20]. However, benzodiazepine was used for postsurgical sedation in most of those studies. As mentioned previously, preanesthetic medication is not administered at our hospital; accordingly, all reported benzodiazepine use was in the form of oral sleeping agents. In addition to the pharmacological action of benzodiazepine, the characteristics of patients who were administered oral benzodiazepine before surgery might have contributed to the classification of oral benzodiazepine use as a risk factor.

The patient's general status upon ICU admission might also have an impact on delirium development. The APACHE II score is widely used to evaluate a patient's general status upon ICU admission. Lescot et al. conducted a cohort study of patients after orthotopic liver transplantation and reported that a high APACHE II score was a risk factor for delirium development [21]; however, no statistically significant difference was observed between patients with high and low APACHE II scores in the present study.

Internal iliac artery embolization may be required during EVAR, depending on the location of the aneurysm or the anatomical form. The original blood flow to the pelvic viscera originates from the inferior mesenteric artery and bilateral internal iliac artery; however, the pelvic viscera must depend on a single blood source when a unilateral internal iliac artery embolization is performed and must depend substantially on blood flow from collateral circulation when a bilateral internal iliac artery embolization is performed. Therefore, embolism may lead to ischemia of an internal organ, resulting in development of ischemic enteritis or buttock claudication, although the incidence range is clinically acceptable [22–24]. Whereas internal iliac artery coil embolization has been considered a risk factor for the development of delirium because it increases the physiological stress level (e.g., ischemia of an internal organ), it was not found to be a risk factor in this study. At our hospital, we preserve blood flow through the unilateral internal iliac artery and do not perform bilateral internal iliac artery embolization in order to reduce the above-mentioned ischemic organ damage. When bilateral internal iliac artery embolization is required, we perform a 2-stage internal iliac artery embolization at a minimum interval of 2 months to enhance the collateral circulation after internal iliac artery embolization. This method may reduce organ damage, contribute to the prevention of ischemic enteritis or buttock claudication, and confer an advantage in terms of preventing delirium development.

The underlying mechanism associated with delirium development remains controversial. In addition to the risk factors examined in this study, several other factors have been suggested to influence delirium [19]. However, very few studies have evaluated the risk factors of delirium after stent-graft placement via EVAR. To the best of our knowledge, this is the first report to investigate delirium after stent-graft placement via EVAR. However, this study was limited by its small sample size and retrospective nature; further studies are needed to confirm our results.

## 5. Conclusion

In this small, single-institution, retrospective study, comorbid renal dysfunction, preoperative benzodiazepine use, and intraoperative transfusion were predictive of developing delirium in the ICU after elective EVAR. Developing delirium was associated with longer hospital stay.

## Figures and Tables

**Table 1:** Characteristics of patients who underwent elective endovascular aortic repair.

Characteristics	Delirium	Nondelirium	Total	*p* value
*N*	20	61	81	
Age (years)	78.7 ± 7.5	73.0 ± 7.6	74.4 ± 7.9	0.006
Male gender	17 (85%)	50 (82%)	67 (83%)	0.756
Left ventricular dysfunction	1 (5%)	4 (7%)	5 (6%)	0.80
Hypertension	16 (80%)	43 (70%)	59 (73%)	0.41
Cerebral infarction	3 (15%)	5 (8%)	8 (10%)	0.38
Diabetes	2 (10%)	2 (3%)	4 (4.9)	0.23
Renal dysfunction	7 (35%)	7 (11%)	14 (17%)	0.016
Preoperative use of benzodiazepine	6 (30%)	4 (6.6%)	10 (12.3%)	0.018

**Table 2:** Clinical parameters and outcomes of patients.

Parameters	Delirium	Nondelirium	Total	*p* value
*N*	20	61	81	
Internal iliac artery coil embolization	3 (15%)	12 (20%)	15 (18%)	0.89
Irradiation time (minutes)	27.4 ± 16.5	24.3 ± 14.8	25.1 ± 15.3	0.372
Amount of contrast media used (mL)	93.8 ± 53.4	89.6 ± 34.4	90.6 ± 41.4	0.767
Surgical time (minutes)	122 ± 41.7	102 ± 37.3	107 ± 38.9	0.047
Intraoperative red blood cell transfusion	4 (20%)	1 (1.6%)	5 (6.1%)	0.003
APACHE II score	13.3 ± 4.2	11.8 ± 3.5	12.3 ± 3.7	0.17
APACHE II score ≥16	7 (35%)	8 (13%)	15 (18%)	0.067
ICU length of stay (days)	3.3 ± 2.4	2.5 ± 0.82	2.7 ± 1.4	0.169
Hospital length of stay (days)	14.5 ± 11.9	9.87 ± 5.31	11.0 ± 7.8	0.019

APACHE II: Acute Physiology and Chronic Health Evaluation II; CI: confidence interval; ICU: intensive care unit; OR: odds ratio.

**Table 3:** Factors associated with the development of delirium in the intensive care unit after elective endovascular aortic repair.

	OR	95% CI	*p* value
Renal dysfunction	4.5	1.15–17.5	0.031
Preoperative use of benzodiazepine	7.63	1.67–34.8	0.009
Intraoperative transfusion	12.3	1.11–134	0.041
